# Assessment of accuracy and recognition of three-dimensional computerized forensic craniofacial reconstruction

**DOI:** 10.1371/journal.pone.0196770

**Published:** 2018-05-02

**Authors:** Geraldo Elias Miranda, Caroline Wilkinson, Mark Roughley, Thiago Leite Beaini, Rodolfo Francisco Haltenhoff Melani

**Affiliations:** 1 Department of Social Dentistry, Laboratory of Forensic Anthropology and Odontology (OFLAB), School of Dentistry, University of São Paulo, São Paulo, Brazil; 2 Face Lab, Liverpool John Moores University, Liverpool, United Kingdom; 3 Department of Preventive and Social Dentistry, Uberlândia Federal University, Uberlândia, Brazil; Seoul National University College of Medicine, REPUBLIC OF KOREA

## Abstract

Facial reconstruction is a technique that aims to reproduce the individual facial characteristics based on interpretation of the skull, with the objective of recognition leading to identification. The aim of this paper was to evaluate the accuracy and recognition level of three-dimensional (3D) computerized forensic craniofacial reconstruction (CCFR) performed in a blind test on open-source software using computed tomography (CT) data from live subjects. Four CCFRs were produced by one of the researchers, who was provided with information concerning the age, sex, and ethnic group of each subject. The CCFRs were produced using Blender^®^ with 3D models obtained from the CT data and templates from the MakeHuman^®^ program. The evaluation of accuracy was carried out in CloudCompare, by geometric comparison of the CCFR to the subject 3D face model (obtained from the CT data). A recognition level was performed using the Picasa^®^ recognition tool with a frontal standardized photography, images of the subject CT face model and the CCFR. Soft-tissue depth and nose, ears and mouth were based on published data, observing Brazilian facial parameters. The results were presented from all the points that form the CCFR model, with an average for each comparison between 63% and 74% with a distance -2.5 ≤ x ≤ 2.5 mm from the skin surface. The average distances were 1.66 to 0.33 mm and greater distances were observed around the eyes, cheeks, mental and zygomatic regions. Two of the four CCFRs were correctly matched by the Picasa^®^ tool. Free software programs are capable of producing 3D CCFRs with plausible levels of accuracy and recognition and therefore indicate their value for use in forensic applications.

## Introduction

Facial reconstruction or facial approximation is a technique that aims to reproduce the individual facial characteristics, prior to death, based on interpretation of the skull, with the objective of recognition leading to an identification [1]. The resulting reconstruction can be published in the media and therefore lead to recognition of the deceased by family or friend and consequently to identification by other means.

Nowadays, there are two techniques to perform a three-dimensional (3D) facial reconstruction: the manual or the computerized. 3D computerized craniofacial forensic reconstructions (CCFR) have some advantages provided by the use of a computer as the enhanced visualization tools allow for the display of bone and the skin together with many transparency adjustments [2]. It is possible to evaluate the reconstruction during the process, correcting mistakes [2, 3]. Other advantages include reduced risk of damaging the skull and the reassembly of skull fragments or the replacement of absent portions [4].

Nevertheless, some disadvantages of the CCFR are also observed. Firstly, some methods can be time consuming [4]. Secondly, many of the existing methodologies, may require a certain level of experience from the operator [4]. Some of these methods have undergone rigorous scientific evaluation of the accuracy and recognition levels [3–7]. There is a growing necessity to determine a clear and reproducible protocol to evaluate the quality of 3D CCFR in relation to the actual face of the subject [4, 8].

In order to overcome these limitations, this research highlights the importance of evaluating the results of facial reconstruction using new software. It is fundamental to clarify that the accuracy of the CCFR is directly related to the prediction of morphological facial traits, while recognition level is related to the capacity of automated recognition [5]. In this paper both of these variables were tested. The evaluation of a CCFR is important in the continuous enhancement of the methods involved, even though existing research presents little or no focus in the evaluation of the result quality achieved [9].

To our knowledge, there is no other research that evaluated the accuracy and recognition level of a CCFR with the software used in this study. With new methods, there is a need to achieve simple, reliable and automated ways to perform this task, returning more realistic images [8]. The 3D CCFR is in current need for verification of the reliability and reproducibility with available tools and others to be developed in the future [5, 6, 10], as there are no computerized tools globally accepted by the forensic community [11].

Therefore this work presents software not often used for forensic means, focusing more on the evaluation of the results than in the technique itself, as program handling can be learned. The use of such open source software might enable a wider cohort of researchers with the opportunity to produce CCFR, making CCFR more simple, efficient and accessible. The aim of this study is to evaluate the accuracy and recognition level of 3D CCFR using free open-source software programs.

## Materials and methods

Four living volunteers donated their existing computer tomography (CT) exams and frontal standardized photographs: **subject 1:** Female, 22yo; **subject 2:** male, 24yo; **subject 3:** female, 49yo; **subject 4:** male, 21yo. They were classified as Brazilian Caucasian by the researcher and had no record of orthodontic treatment, craniofacial, plastic surgery or facial deformity. Written informed consent was obtained from all subjects and no subject was exposed to radiation for the purposes of this research. The Ethics committee of the University of São Paulo—Brazil, under protocol number 1.608.387, approved the procedures. The individual in this manuscript has given written informed consent (as outlined in PLOS consent form) to publish these case details.

The DICOM files (digital imaging and communications in medicine) from the CT data were first reconstructed in the open source software Horos^®^ 3.0 version (www.horosproject.org), a viewer that can export stereolithographic (STL) files from selected surfaces, such as bone or skin tissues by selecting the respective density of the region of interest. The generated STL file was imported into Blender^®^ 2.78 version (www.blender.org), a free and open-source 3D modeling and animation software where the CCFRs were performed.

**Fig 1:**
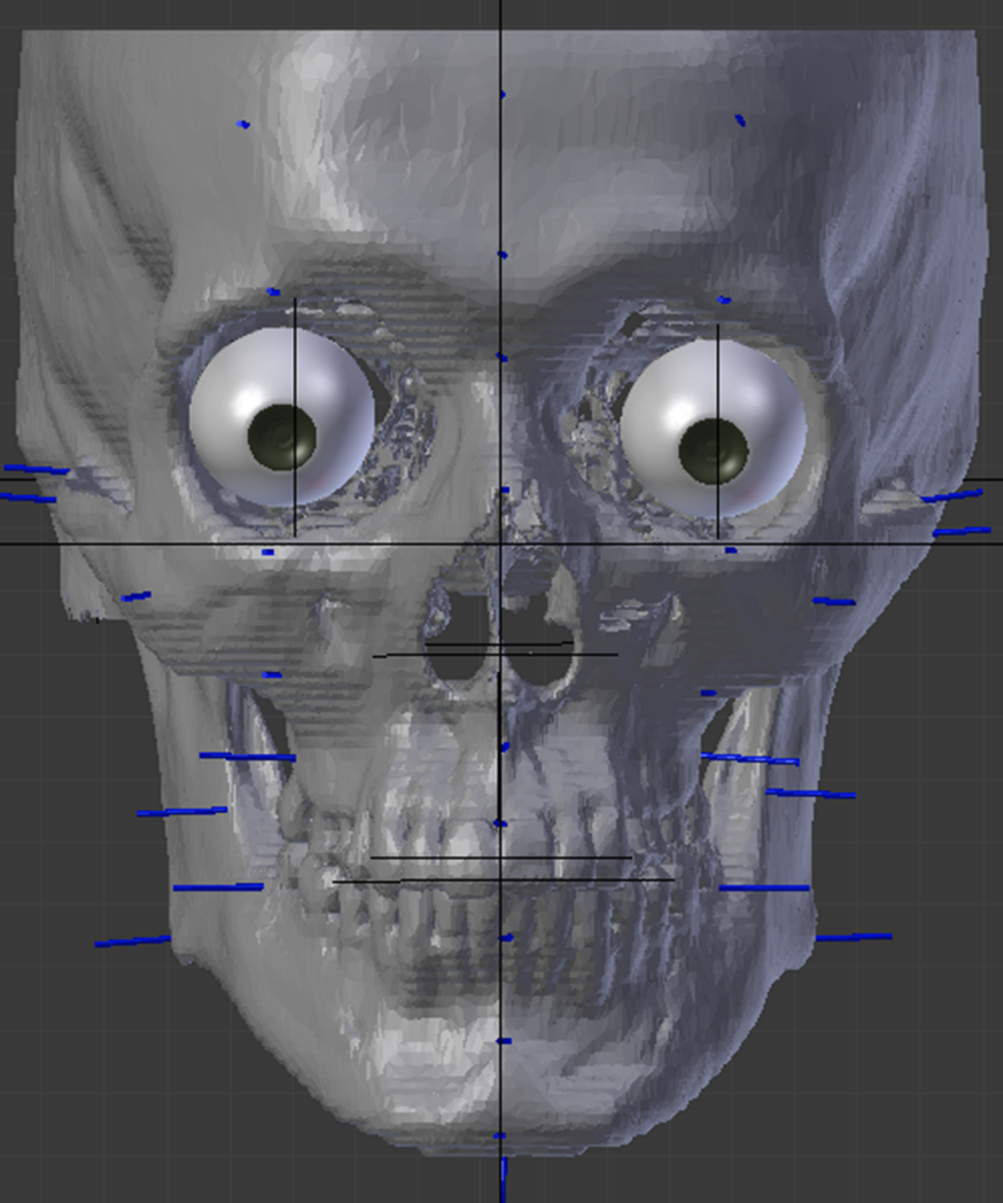
Soft tissue markers placement, eyes, mouth and nose guides.

The skull was positioned in reference to the Frankfurt plane and soft tissue markers were placed over its surface, using landmarks as described in a study of a Brazilian sample [1], that reports a table with male and female values for 10 mid-sagittal points and 11 bilateral points in a total of 32 references (Fig 1).

The shape and position of the eyes, nose and mouth also followed pre-established parameters:

- Eyes: eyeballs of 24mm in diameters were placed inside the orbital cavity so that the eyeball and pupil were centrally located within the orbits, 4 mm from the roof and 4–5 mm from the lateral wall [12]. Protrusion was established as tangents from the margins of the orbital cavity crossing only the iris portion of the eye [13]. The endocanthus of the eye, was placed 2 mm lateral to the lacrimal bone crest at its middle and the exocanthus approximately 3–4 mm medially from the malar tubercle [12]. For the CCFR’s, the eyelids were modeled closed as the CT exams were produced in the same position.
- Nose: the maximum width of the piriform cavity represents approximately 3/5 of the maximum width of the nose [14]. The tip of the nose was estimated to be at the crossing point of two lines: the nasal bone projection and a perpendicular line from the prosthion projection, in a 90° [15].
- Mouth: The inter-canine distance is estimated as 75% of the total mouth width [16]. The height of the lips vermillion is 26% of the width [17]. The lower and upper lip intersection should be placed in the lower section of the upper central incisor [18].

**Fig 2:**
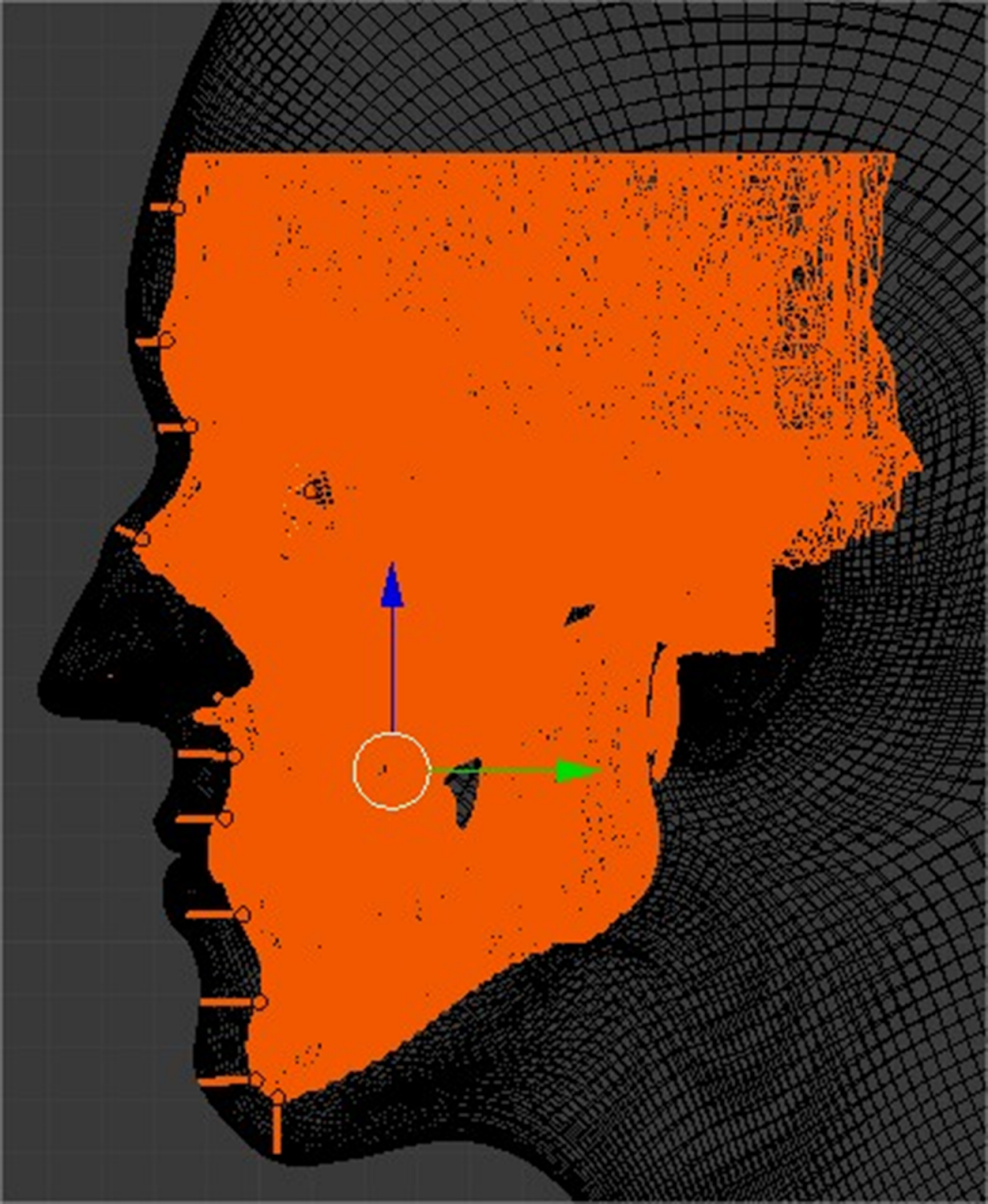
Template placement over the soft-tissue markers in lateral view.

In a forensic investigation, the anthropological examination will estimate the age, sex and ethnic group of the human remains. It is through this information that a 3D face model template was selected as appropriate on MakeHuman^®^ 1.1.0 (makehuman.org). MakeHuman^®^ is a 3D software with the goal to create humanoid models. The chosen template generated by the software was exported as an STL files and imported into Blender^®^ and placed over the skull model to be reconstructed (Fig 2). The CCFRs were reconstructed as a blind study by one of the researchers, with the only known information as age, sex and ethnic group of the individual.

The addition of individual characterization was kept to a minimum due to the fact that incorrect skin color, hair and other modifiers can impair identification and lead to error [6, 19]. This research followed the recommendation of authors of existing CCFR research and the CCFR was presented without hair, the skin was left in a shade of gray and the eyes were closed.

**Fig 3:**
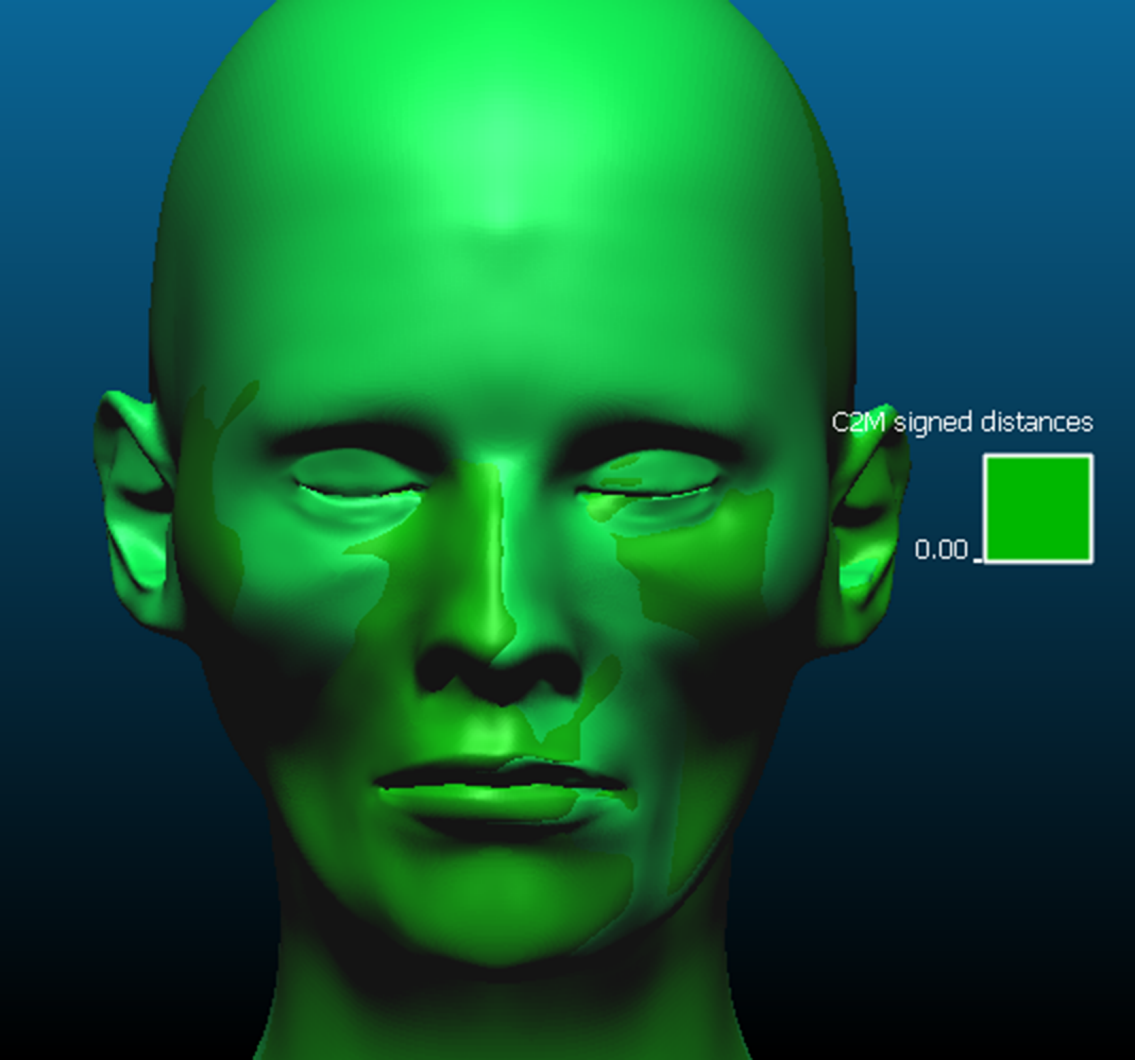
Alignment test.

The quantitative evaluation of the accuracy was carried out using CloudCompare^®^ 2.6.3 software (www.cloudcompare.org), where two 3D point clouds, each corresponding to triangular faced mesh models, can be compared. To complete this task, the CT soft tissue model was placed as “reference” and the reconstruction as “compared” and the software calculated the distances between the two point clouds. An important step is to align the models, as described by Decker (2013) [20] using the nasion, and the deepest lateral points of the orbits as reference. Sequentially, one can use the “cut” tool to eliminate any lateral or lower parts that could be observed only in one of the models. Prior to attempting to align the CCFR 3D model with the respective CT 3D model, a test alignment with two identical models took place to verify the method of alignment. If the match was different to 100%, the method of alignment would be incorrect as the two 3D models were identical. (Fig 3). Therefore it was possible to calculate the discrepancy in millimeters between the meshes, which have minimum and maximum values, graphics, histograms and numerical tables as outputs and also can be observed through a color-map applied on the compared model’s surface.

The evaluation of the automated recognition level of the CCFR was performed using Picasa^®^ 3.9 (https://picasa.google.com/), which is software for image storage. In the field “people” the standardized frontal photographs of each subject were inserted and named. Previously the frontal images captured from the CT soft-tissue model and from the CCFR were inserted into the data-base. Once the “people” field is calibrated by the imported images, the program can automatically scan existent ones. Consequently one of the four events occurs: 1- the image is not recognized as a face; 2 the image is recognized as a face but not a matching to any “people” (classified as unnamed); 3- the image is correlated to a known person and confirmation may be asked; 4- the image is incorrectly matched to the wrong person.

## Results

**Fig 4:**
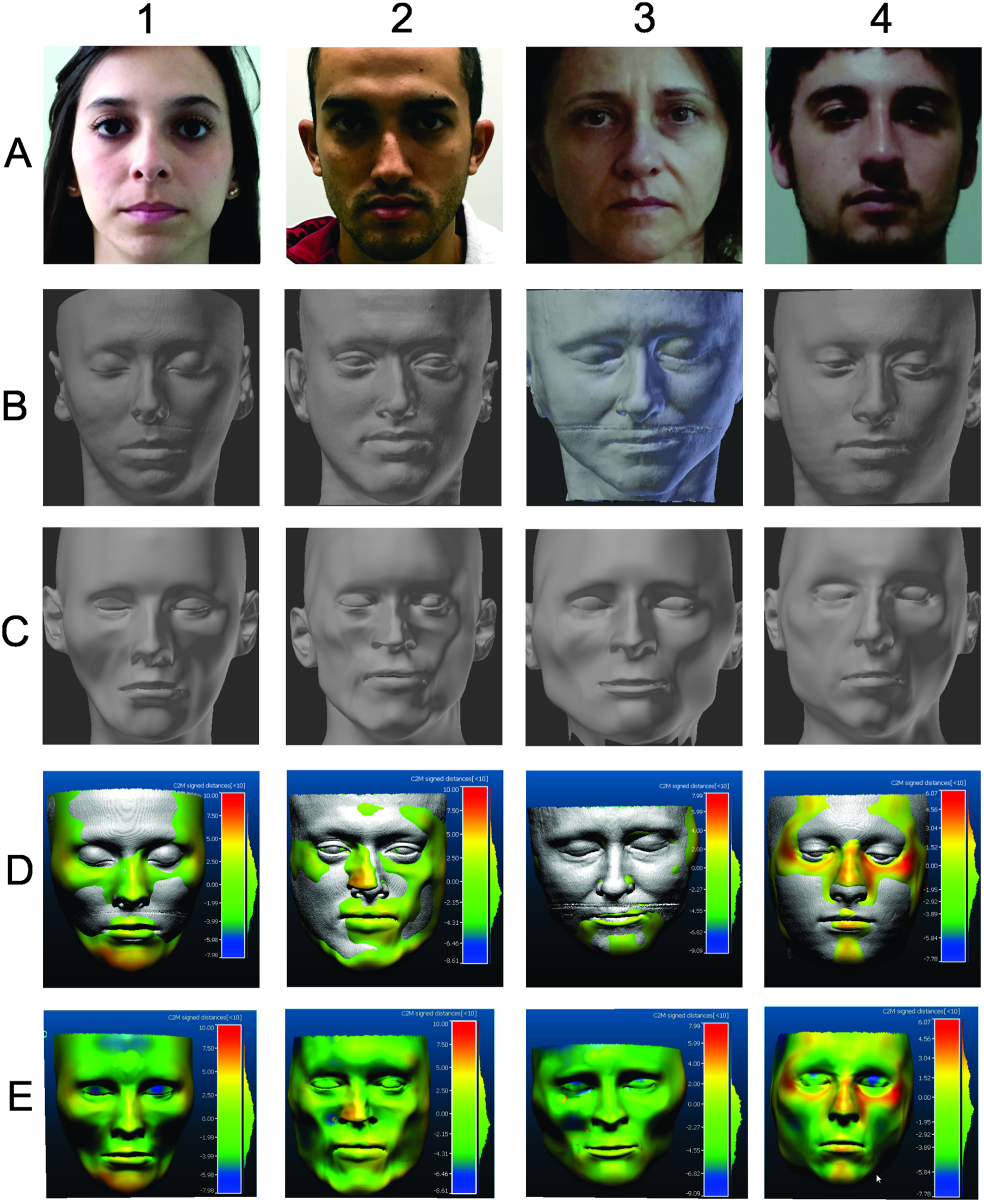
The columns show subjects 1, 2, 3, 4. Line A: photograph; line B: CT surface; line C: reconstruction; line D: result of the comparison between CT and reconstruction with the respective color map. E: reconstruction with color map.

The CloudCompare^®^ showed the discrepancy (mm) between the surface of the CCFR and the surface of the CT face model. The positive numbers indicated that the surface of the reconstruction was overestimated in relation to the real face, while the negative numbers indicated that the surface was underestimated (Fig 4). The color map generated shows green when there is a distance of less than ± 2.5mm between the meshes, the colors that vary from yellow to red show when the distance is between 2.5mm to 10mm, and the blue to the dark blue colors show when the distance is -2.5mm to -10mm. The grey color areas are representative of the CT 3D model. In Fig 4D the CT 3D model and the CCFR 3D model are aligned on top of each other, overlapping. When the CT 3D model appears (the grey colour) it means that the surface of the produced CCFR 3D model was underestimated (Fig 4D). When analyzing each face region it can be noted that in all cases the greatest discrepancies occurred in the cheek and in the eyes, which were underestimated (blue), while the chin and zygomatic area were overestimated (red) (Fig 4E).

The results were analyzed through descriptive statistics using the average, standard deviation and maximum and minimum deviation (mm) between the surface of the CCFR and the CT face model (Table 1). Table 2 shows the distribution (%) of the error deviation ± 2.5 mm, as performed by Lee et al [21].

**Table 1:** Average and standard deviation of the discrepancy between the facial surface of the reconstruction and corresponding subject.

	Subject A	Subject B	Subject C	Subject D
**Average +/- (mm)**	0.33	-0.10	-1.66	-0.42
**Standard Deviation (mm)**	2.78	2.62	2.36	2.28
**Maximum Upper/Lower Deviation +/- (mm)**	9.29/-7.90	9.89/-8.61	7.99/-9.02	6.07/ -7.70

**Table 2:** Distribution (%) of the deviation error between the surfaces of the reconstruction and the subject within each defined range (2.5 mm).

	Subject A	Subject B	Subject C	Subject D
Deviation range (mm)				
** -10.0 ≤ X <- 5.0**	1.70	1.20	9.82	2.37
** -5.0 ≤ X < -2.5**	13.87	17.39	22.93	13.90
** -2.5 ≤ X ≤ 2.5**	63.21	63.61	64.68	73.68
** 2.5 < X ≤ 5**	16.01	14.47	2.17	9.51
** 5 < X ≤ 10**	5.21	3.32	0.40	0.54
** Total (%)**	**100**	**100**	**100**	**100**

**Fig 5:**
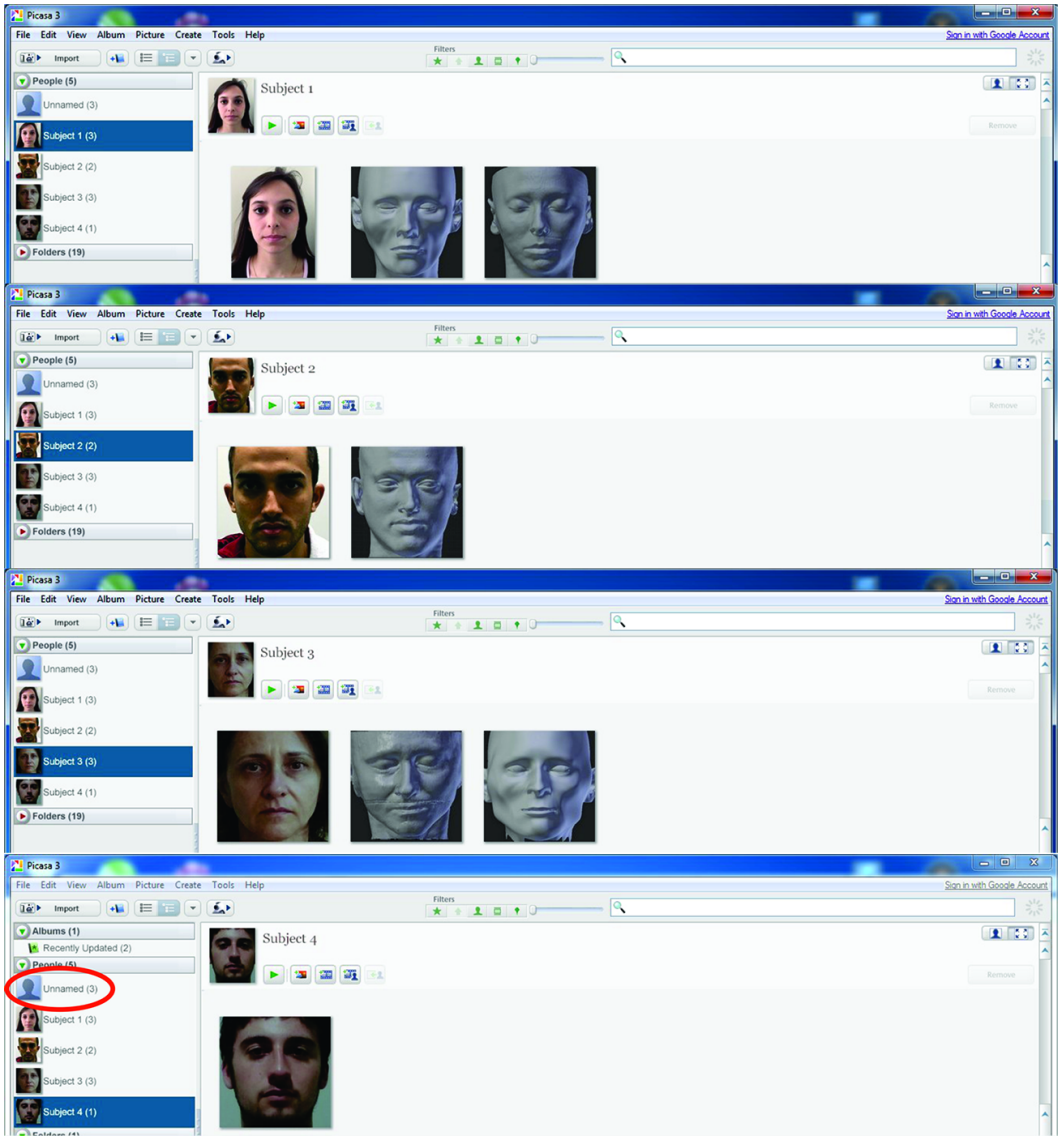
Analysis of the recognition level of photographs, CT and CCFR in Picasa^®^. The CCFRs of subjects 2 and 4 plus the CT of subject 4 were classified as unnamed (circle).

When evaluating the recognition level in Picasa^®^ the program recognized 100% of imported images as a face. In addition, the program correctly assigned all photographs to the respective subjects, three of the four CTs (subjects 1, 2 and 3) and two of the four CCFRs (subjects 1 and 3) (Fig 5).

## Discussion

The progress of computing and the improvement of imaging techniques in recent years has promoted the development of fast and flexible programs that can be used for CCFR [2, 4]. However, it is very important that researchers analyze the accuracy, reliability and reproducibility of these programs [2, 4] so that CCFR becomes accepted in the forensic field [4]. In addition, a constant reevaluation will promote improvement and increase reliability [4].

A critical element in the design of a CCFR is the evaluation of its accuracy. One-to-one comparison and face pools are useful methods for assessing CCFR accuracy, but other objective methods are required to assess quantitative accuracy, especially due to increased application of CCFR production methods [21]. The evaluation through the comparison of the geometric surface is an effective tool to evaluate the accuracy of the CCFR [21]. Two 3D facial models can be aligned so that differences in facial contour and reconstruction can be numerically computed, as well as providing a spatial map of the differences of each facial region reconstructed [2, 3, 21, 22]. Thus a quantitative evaluation of reconstruction error can be performed, observing three-dimensional surface differences [2, 3].

This was the method used in this research to evaluate the accuracy of the CCFR. For a more objective and quantitative evaluation, the CloudCompare^®^ software was used, since the program quantitatively evaluates the morphological discrepancy of the surface between the reconstructed face and the subject. The purpose of these comparisons was not to quantify the similarity, but to measure the accuracy of the approximation via metric comparison [20]. Short et al. [23] evaluated the difference between landmarks, but the problem is that the program measures the distance between the closest points and that are not necessarily the landmarks. This was not a problem in this research since the distance between the CCFR landmarks and the real face was not evaluated [23], but the distance between the meshes in a holistic way.

The entire reconstruction was not analyzed, as the posterior region of the head, ears and below the mandible were removed, as was carried out by other authors [21, 23]. The results showed that the mean distance found in this study varied from -1.66 to 0.33mm (Table 1). Similar results were found in the literature with a mean error varying between 1.14mm [22]; -0.49 to -0.31mm [5]; -0.2 to 0.4mm [21] and 4.0mm [11].

When the total points of each reconstruction were analyzed, 63.20% to 73.67% presented a distance within the range of -2.5 ≤ x ≤ 2.5 mm between the CCFR (compared) and the real face (reference) (Table 2). Similar results were described in previous studies with percentages varying from 54% to 76% [5], 52% to 60% [7], 79% to 87% [21] e 56 to 90% [23]; when an error of ± 2.5mm was applied using a sample of three, two, three and ten subjects, respectively. The soft tissue thickness of an individual will never be completely accurate since the tissue thicknesses markers employed are averages [23]. In addition, minor errors may occur when converting CT files for viewing and exporting to programs [21, 24]. Another factor that influences the success of the reconstruction is the lack of information that BMI has on soft tissue thickness [20]. The level of precision required for a facial reconstruction to result in recognition is not clearly known [11]. Laboratory studies suggest that it is not possible to produce an exact portrait, but it should be possible to estimate the facial morphology with enough accuracy to allow recognition [10]. Therefore, the result of the accuracy found in this research is similar to other studies and can indicate value for use in forensic investigations.

Accuracy assessment also assists in the analysis of specific regions of the face. Prediction of location, size and morphology of facial features—eyes, nose, mouth and ears—is critical to the accuracy of CCFRs [21]. Some measures of the anatomical regions of the reconstruction are underestimated (they are smaller compared to the target model) and other areas are overestimated (larger than the target) [23]. When evaluating each region of the face, it can be observed that in all cases the greatest discrepancies occurred in the cheek and in the eyes that were underestimated (blue), while the chin and the zygomatic area were overestimated (red). This demonstrates that these regions of the CCFRs in this research need to be improved. Some variation in the cheek was already expected due to the gravitational detachment caused in the patient lying supine position in the CT [20, 22, 25]. In addition, the soft tissue thickness markers utilized in this research comes from cadavers, so thanatological effects may have influenced the accuracy of the reconstructions since of the four subjects, three had a negative average (Table 1). Any measure taken after death is slightly reduced and the position of features may be subject to high levels of post-mortem change [25]. This can be verified by replicating this work and using different tables of soft tissue thickness to evaluate which one presents better accuracy.

When comparing these results with the literature it was observed that the studies differ when specific areas of the face are analyzed, due to different methodologies and software used. These differences may also be the result of positional effects when performing CT scans making unmatched facial features, for example, facial expression, mouth and open eyes.

One study observed that, on average, the area of the chin was the one with the greatest similarity and the area of the eyes smaller [9]. Guyomarc’h et al (2014) found a greater error in the region of the ear (7mm), moderate in the mouth (4.5mm) and smaller in the nasal regions (3.1mm) and eyes (2.9mm) [11]. The reconstructions had larger cheeks, more prominent upper lip and a pattern of error in the ear, and the ear and tip of the nose were the areas with greater error (> 5 mm) [7].

Decker [20] carried out four reconstructions of the same individual, two being computerized using the FaceIT and ReFace programs and two manual clay craniofacial reconstructions and found out that there was great variation between the reconstruction methods [20]. All techniques performed well on nasal width, but there was variability in the angle / length of the nose, underestimating the width of the mouth, glabella and chin [20]. Another study showed that the nose and mouth were the most difficult areas to reconstruct [24]. The nose and mouth areas were overestimated with the biggest difference in the nose (7mm) [23]. In CCFR of subject 2 there was a positive difference on the right side and negative on the left side of the nose, which may have contributed to its non-recognition by the program.

The accuracy of facial reconstruction may directly affect the success of recognition [23] since the ultimate goal of reconstruction is not accuracy, but the recognition and subsequent success of identification [2, 3, 22]. Thus, recognition performance seems to be the most appropriate way of evaluating its effectiveness [19]. The automated recognition test consists of comparing the result of the CCFR with a candidate base including the actual person [2, 3]. The most valuable validation of reconstruction techniques is still based on human recognition, because it has a more practical relevance [24]. The majority of facial reconstruction studies evaluate the recognition level subjectively (examiners).

However, the future of facial recognition especially when there is a large base of images undoubtedly depends on facial recognition software that is far less cumbersome and more efficient than having human reviewers [26]. Thus for a more objective result the evaluation of recognition of the reconstructions was carried out by a program called Picasa^®^, a facial recognition software. The program correctly assigned all the photographs to the respective subjects, three of the four CTs (subjects 1, 2 and 3) and two of the four CCFRs (subjects 1 and 3) (Fig 5). Although all reconstructions have a close error rate (Table 2), several factors may have contributed to non-recognition by the program. For example, subjects 2 and 4 are with eyes open in CT and closed in CCFR, difference in nose, etc. A study showed that Picasa^®^ was able to recognize 100% photographs and 27.5% of CT [26]. However, the program had not yet been used for CCFR recognition as performed in this work. Moreover, it was difficult to compare this similarity test with other studies since most of them use the subjective "pool face" methodology and the results vary widely. New researches evaluating the similarity of CCFRs using computational programs and with a larger sample are necessary.

Computational systems are dependent on facial templates, mean tissue depth markers, and specific population data [2, 27]. In this study, soft tissue thickness markers [1], lip prediction [17] and nose tip prediction methods [15] were used from studies with Brazilians in order to apply regional measures for better reconstruction efficiency [21, 24, 27].

The first step in reconstruction is the anthropological examination of the skull to estimate age, sex, and ancestry. It is through the anthropological examination that the 3D face model template will be chosen to be used in the CCFR. The template specification will strongly influence the final result, if an inappropriate template is chosen, bias may occur [2, 3, 8, 24]. Using only a generic template the potential to produce a biased model is high [2, 3]. When the difference between the 3D face model template and the target skull is large, the required deformation will be more pronounced, which can result in an implausible, unrealistic or cartoon-like facial reconstructions [24].

The performance of the reconstruction depends on the quality of the database used. More precisely, successful CCFRs can depend on the available CT scan data in a database that represents any skull of a given population [25]. Images in a database from CT provide good definitions of structures. However, this imaging technique is invasive and for ethical and legal reasons it is difficult to build a large database of healthy people [25]. Templates of 3D face models from the MakeHuman^®^ database were used in this project as it was possible to obtain an appropriate template for the anthropological profile of the skull to be reconstructed. So the problem of a limited template base [4] has been eliminated.

One of the disadvantages of using CT is the sensitivity to amalgam and metal dental restorations that produce artifacts in the images produced during the CT exam [3]. This research focuses on CCFRs of living individuals however, with CCFRs of deceased individuals, photogrammetry can be used to obtain the 3D model of the skull from photographs. However photogrammetry does not show internal regions of the skull that are important for other forensic analysis. One study obtained a 3D skull model through photogrammetry and subsequently performed the reconstructions in Blender^®^ [28]. The authors highlighted some advantages such as the ease of visualization, storage and sharing of the reconstruction [28]. The technique presented here also allows remote access and so it is possible to work with experts who are not in the same physical environment, avoiding the transport of the remains.

Turner et al. [29] developed a computer system called ReFace, a program that uses a dense placement of landmarks rather than the placement of sparse landmarks. The problem is that ReFace is an automated program developed by the Federal Bureau of Investigation (FBI) and is not available to the scientific community, so it cannot be used by other experts or be tested by other researchers.

All software programs used in this research study are free and open source, which can be downloaded and used by anyone. The use of free software eliminates the initial cost and contributes to the dissemination of the technique. Most CCFR methods make use of non-rigid generic deformation, which is mathematically well defined and easy to use but if not used correctly they can deform the face presenting it in an unrealistic way [3]. Another disadvantage of free software is that they can be disabled by the developer leaving the user unsupported or without updates.

## Conclusion

The results from this study demonstrate that free open-source software programs are capable of producing 3D computerized forensic craniofacial reconstructions with plausible level of accuracy and recognition and therefore indicate value for use in forensic investigations. Subsequent studies are required using different tissue thickness and with a larger sample or using alternative software programs to evaluate the method further.
